# Alcohol use in the prehospital setting: a diagnostic challenge in patients treated by a physician staffed mobile intensive care unit

**DOI:** 10.1186/s40064-016-2921-y

**Published:** 2016-08-03

**Authors:** Terhi Kauppila, Janne Virta, Leena Lindgren, Ilkka Virkkunen, Antti Kämäräinen

**Affiliations:** 1 School of Medicine, University of Tampere, 33014 Tampere, Finland; 2 Emergency Medical Services, Tampere University Hospital, Tays, PL2000, Tampere, Finland; 3 FinnHEMS R&D, WTC Helsinki Airport, Lentäjäntie 3, 01530 Vantaa, Finland

**Keywords:** Alcohol, Diagnostics, Intoxication, Prehospital, Out-of-hospital, Emergency

## Abstract

**Background:**

Alcohol use among emergency patients has been studied earlier, but the data regarding alcohol use especially among critically ill and injured patients treated in the prehospital setting is scarce. The aim of this study was to evaluate the incidence of alcohol use and the characteristics of cases attended by a physician staffed mobile intensive care unit (MICU).

**Findings:**

During a 2 month period, exhaled air alcohol concentration—measured as a part of routine patient examination in all adolescent and adult patients treated by the MICU—was recorded. The MICU encountered 258 patients, of which 82 could be tested for alcohol use. Of the tested patients 43 % gave a positive breath test result. Proportion of male patients providing a positive result in the breath test did not differ significantly those of women. The primary reason for not to test the patient was a decreased level of consciousness in one-fifth of the initial 258 patients.

**Conclusions:**

A significant proportion (47 %) of the encountered patients could not be tested due to their critical condition. Alcohol use was observed in 43 % of those capable of providing a breath test sample. The rate of positive tests seemed to be higher than those reported from emergency departments. Novel diagnostic methods to detect alcohol consumption in non-cooperative patients are warranted.

## Background

Alcohol use among emergency department patients has been studied for over 30 years. In a study by Holt et al. 40 % of patients attending the emergency department of a large teaching hospital were under the influence of alcohol and the majority of them were men. A positive breath test as an indicator of alcohol use was more common in cases of self-poisoning, head injury and major trauma than in general medical or surgical patients. Furthermore, 82 % of patients presenting with a lowered level of consciousness gave a positive result in the breath test (Holt et al. 1980). In another study, alcohol use was verified in approximately one-third of trauma patients of working age admitted to a casualty department of a university hospital (Antti-Poika and Karaharju 1986). As alcohol use predisposes to injuries and repeated use of emergency department (ED) services, it is important that alcoholics and heavy consumers are identified by health care personnel (Antti-Poika and Karaharju 1986; Cherpitel et al. 1996). However, the identification of a drinking problem has proven difficult and e.g. clinical signs such as slurred speech and abnormal co-ordination unreliable indicators of alcohol use (Holt et al. 1980; Cherpitel et al. 1996).

As a clinical aspect in an emergency, alcohol use of the patient could affect e.g. the evaluation of traumatic brain injury (Shahin et al. 2010), the delivery of anaesthesia (Fassoulaki et al. 1993) or drug metabolism (Weathermon and Crabb 1999) regardless of the treatment setting.

Despite reported data on alcohol use among ED patients, data regarding patients treated in the prehospital setting is scarce. Furthermore, to our knowledge no reports have been published on the topic of alcohol use in context of the most critical prehospital emergencies. The purpose of this prospective study was to evaluate the incidence of alcohol use using a breath analyzer, the feasibility of the breath test analysis itself, as well as patient and mission characteristics of cases attended by a physician staffed mobile intensive care unit (MICU) during a two month period.

## Methods

The study was conducted between 7th April and 8th June 2012 in the physician staffed MICU of Pirkanmaa county, Finland, covering a population of 486,000. The MICU, staffed with an anaesthesiologist and a paramedic, responds to the most critical emergencies such as presumed cardiac arrest and high energy trauma. As a part of routine patient examination, exhaled air alcohol concentration is measured in all adolescent and adult patients treated by the MICU—children under the age of 15 were excluded from the study. During the study period, the results of this test were prospectively recorded from the MICU run sheets and mission database. Additionally, data on patient age, gender, medical condition and the type of emergency were recorded.

Alcohol use was examined out of exhaled breath by using a single breath alcometer tester (Dräger Alcotest 3000, Dräger Safety AG, Lübeck, Germany). If the tester could not be used, the reason for this was recorded in the MICU run sheet. As there was no new treatment or examination modality involved in the study setting, and by the permission of the Department of Science Centre of Pirkanmaa Hospital District, the requirement for patient consent and approval of the regional ethics committee were waived. Statistical analysis was performed using the IBM SPSS Statistics version 20.0. (IBM, Armonk, NY, USA) with statistical significance set at p < 0.05.

## Results

During the study period, the MICU encountered 258 patients, out of whom 161 (62 %) were men and 97 (38 %) women. The mean age of the patients was 51 years (range 0–92). The most common types of emergency were classified as; blunt trauma (n = 48, 19 %), neurological cause (excluding presumed ischemic stroke) (n = 43, 17 %), cardiac arrest (n = 34, 13 %) and intoxication (n = 32, 12 %).

Of the encountered patients, 82 (32 %) patients, 52 men (63 %) and 30 women (37 %), could be tested with the alcometer. The primary reason for failed testing was decreased level of consciousness in one-fifth (n = 56, 22 %) of the initial 258 patients. Furthermore, 38 (15 %) patients were in cardiac arrest, and 26 (10 %) patients were unable to perform the breath test due to shortness of breath or fatigue. Five patients (2 %) refused the breath test. Of the encountered patients, 16 (6 %) were children <15 years and thus not tested. Also, the breath test was forgotten or waived due to an unknown reason by the MICU staff in 35 (14 %) cases.

Out of the 82 patients included in the final analysis, 35 (43 %) gave a positive breath test result. The characteristics of these patients are described in Table 1. The proportion of male patients providing a positive result in the breath test did not differ significantly those of women—24 out of 52 men versus 11 out of 30 women, two tailed Fisher exact test 0.489. Also, there was no significant difference when comparing the type of emergency between those with a positive versus with a negative breath test result.

**Table 1:** Alcohol breath test analysis results, characteristics of patients and emergency types

Patients by test result or performance n = 258	Positive result 35 (100 %)	Negative result 47 (100 %)	No breath test 176 (100 %)
Alcohol concentration in the breath test (‰)^a^			Reason for failed testing Impaired consciousness 56 (22 %)
<1	1 (3 %)	NA	Cardiac arrest 38 (15 %)
1–2	14 (40 %)	NA	Shortness of breath 26 (10 %)
2–3	14 (40 %)	NA	Pediatric 16 (6 %)
3–4	5 (14 %)	NA	Refused 5 (2 %)
>4	1 (3 %)	NA	Unknown 35 (14 %)
Patient characteristics			
Male	24 (69 %)	27 (57 %)	108 (61 %)
Female	11 (31 %)	20 (43 %)	68 (39 %)
Age (years)			
<15	NA	NA	16 (9 %)
15–20	2 (6 %)	5 (11 %)	6 (3 %)
21–30	9 (27 %)	8 (17 %)	13 (7 %)
31–40	7 (20 %)	4 (9 %)	11 (6 %)
41–50	8 (24 %)	7 (15 %)	24 (14 %)
51–60	6 (17 %)	6 (13 %)	25 (14 %)
>60	3 (6 %)	17 (36 %)	81 (46 %)
Type of emergency			
Blunt trauma	11 (31 %)	8 (17 %)	29 (16 %)
Intoxication incl. substance abuse	11 (31 %)	5 (11 %)	15 (9 %)
Penetrating trauma	5 (14 %)	3 (6 %)	2 (1 %)
Neurological (excluding ischaemic stroke)	4 (11 %)	8 (17 %)	31 (18 %)
Respiratory	2 (6 %)	1 (2 %)	19 (11 %)
Other	2 (6 %)	22 (47 %)	80 (45 %)

*NA* not applicapble

^a^ = 1 ‰ equals 1000 mg/l or 21.7 mmol/l of ethyl alcohol in blood

Of the 56 patients that could not be tested due to lowered level of consciousness, 19 (34 %) presented with a Glasgow Coma Scale (GCS) (Teasdale and Jennett 1974) score of 3, 23 (41 %) scored 4–8 GCS points and 14 (25 %) patients 10–13 points.

Due to the low rate of breath tests performed in the prehospital setting, we performed a post hoc analysis of the blood alcohol concentrations measured in the receiving emergency department. Of the 160 adult patients in whom the breath test was not performed, 130 were transported to hospital, wherein blood alcohol concentration was measured in 28 patients. Of these patients, 16 provided a negative sample, whereas 12 a positive one (median 57 mmol/l, range 17–111 mmol/l).

## Discussion

In this evaluation of prehospital alcohol use among patients treated by a MICU, three key findings were identified. First, a significant proportion (47 %) of the encountered patients could not be tested using a breath test analyzer due to critical illness or injury. Although non-invasive and of good correlation with blood alcohol tests, the breath test method requires co-operation from the patient (Derogis et al. 1995). Therefore accurate diagnostic methods to detect alcohol consumption e.g. in unconscious patients are warranted, such that are already available for testing of illicit substances (Pehrsson et al. 2011).

Secondly, of the tested patients, alcohol use was observed in 43 % of cases which is slightly higher than the rates reported in studies from emergency departments (Holt et al. 1980, Antti-Poika and Karaharju 1986). Thirdly, although the proportion of men in the whole patient material was higher, there was no significant difference between men and women in regard to the rate of patients providing a positive result in the breath test.

To our knowledge, this is the first prospective study evaluating the incidence and characteristics of alcohol use among emergency medical service (EMS) patients treated by a physician staffed MICU, thus representing a population of patients including the most critical medical and surgical emergencies. Compared to other EMS systems, the observed rate of alcohol use (43 %) was higher than reported from Alaska by Kriegsman and Anthes (28 %) (1998), similar to that reported by McLaughlin (2010) from a Midwestern college town (45 %) and lower than reported from Zurich, Switzerland (73 %), (Holzer et al. 2012). It is likely that more cases of alcohol use could have been identified with diagnostic tools feasible for use in the context of lowered consciousness—the post hoc blood results analysis resulted in further 12 positive test results.

The observed differences in comparison to previous studies from accident and emergency departments may reflect the policy that not all patients encountered by the EMS are transported to hospital, but may also be transported to shelter care or Police custody or even left at the scene (Ross et al. 2013). Also, due to the nature of the critical illness itself and ongoing intensive care, in this patient population harmful alcohol use is frequently unidentified (Tomlinson et al. 2012) in the hospital. Furthermore, the data from hospital settings do not reflect the burden of alcohol use on EMS (Kriegsman and Anthes 1998). However, it is necessary to remark that not all alcohol use can be categorically defined as harmful per se. In Finland, the rate of harmful alcohol use has been defined to be equal or exceed seven daily or 24 weekly alcoholic beverages for men and 5 daily or 16 weekly alcoholic beverages for women. It has been estimated that approximately 500,000 inhabitants exceed these consumption rates in Finland (Halme et al. 2008). As another indicator of harmful alcohol use, driving under the influence (DUI) of alcohol is punishable by law in Finland if the alcohol concentration in exhaled air exceeds 0.5 ‰ or 0.22 mg/l. With these indicators in mind, it can be observed that all but one patient with a positive test result provided either a breath analysis or blood sample result that exceeded the minimum punishable DUI limit.

This study carries weaknesses. First, the eventual sample size of patients performing a breath test was small. This, however, reflects the unsuitability of the breath test method in critically ill patients. Second, the study was conducted in a physician staffed MICU, which responds only to the most critical prehospital emergencies. Therefore this material can be regarded as selected to focus on the critically ill or injured. Still—in contrast to previous studies—all types of emergency and adult patient age groups were included in a prospective manner, expanding the insight to the extent of alcohol use among prehospital patients. Finally, the degree of alcohol intoxications or alcohol use in general were not confirmed with blood tests in the whole patient population, as the focus of the study was limited to the prehospital setting. Overall, based on these shortcomings in data collection the conclusions on prevalence and comparisons to previous reports and other methods of alcohol use detection need to be careful.

From an ethical viewpoint, it could be debated whether routine breath testing for alcohol is justified. Dunham and Chirichella (2012) have approached the subject previously and concluded that testing is justified at least in the most severely injured patients. Also, in an emergency setting, alcohol use of the patient could affect e.g. the evaluation of traumatic brain injury (Shahin et al. 2010), the delivery of anaesthesia (Fassoulaki et al. 1993) by the prehospital or emergency department physician, or drug metabolism (Weathermon and Crabb 1999). In prehospital patients with persisting impaired level of consciousness mortality is considerable (Björkman et al. 2015) and early diagnosis and treatment of the cause is important—such as ruling out alcohol intoxication. It has been previously recognized that emergency department patients do not spontaneously report alcohol abuse themselves (Richoux et al. 2011) and therefore—due to patient safety—we continue to find the method of routine testing to be appropriate at least in the most severely injured or ill patients treated by definition by the physician staffed MICU.

In conclusion, in patients to whom a physician staffed MICU was dispatched due to a presumed high-risk emergency, alcohol use was observed in 43 % of those capable of providing a breath test sample. Importantly, almost half of (47 %) of the encountered patients could not be tested due to a critical condition. Novel diagnostic methods to quantitatively detect alcohol consumption in non-cooperative patients and further studies on the burden of alcohol use on the emergency medical services are necessitated.
